# Clinical, Diagnostic, and Treatment Characteristics of *SDHA*-Related Metastatic Pheochromocytoma and Paraganglioma

**DOI:** 10.3389/fonc.2019.00053

**Published:** 2019-02-22

**Authors:** Abhishek Jha, Kristine de Luna, Charlene Ann Balili, Corina Millo, Cecilia Angela Paraiso, Alexander Ling, Melissa K. Gonzales, Bruna Viana, Rami Alrezk, Karen T. Adams, Isabel Tena, Alice Chen, Jiri Neuzil, Margarita Raygada, Electron Kebebew, David Taieb, M. Sue O'Dorisio, Thomas O'Dorisio, Ali Cahid Civelek, Constantine A. Stratakis, Leilani Mercado-Asis, Karel Pacak

**Affiliations:** ^1^Section on Medical Neuroendocrinology, Eunice Kennedy Shriver National Institute of Child Health and Human Development, National Institutes of Health, Bethesda, MD, United States; ^2^Section of Diabetes, Endocrinology and Metabolism, Department of Medicine, University of Santo Tomas Hospital, Manila, Philippines; ^3^Positron Emission Tomography Department, Warren Grant Magnuson Clinical Center, National Institutes of Health, Bethesda, MD, United States; ^4^Radiology and Imaging Sciences, Warren Grant Magnuson Clinical Center, National Institutes of Health, Bethesda, MD, United States; ^5^Clinical Endocrine Section, National Institute of Diabetes and Digestive and Kidney Diseases, National Institutes of Health, Bethesda, MD, United States; ^6^Division of Cancer Treatment and Diagnosis, National Cancer Institute, National Institutes of Health, Bethesda, MD, United States; ^7^Mitochondria, Apoptosis and Cancer Research Group, School of Medical Science, Menzies Health Institute Queensland, Griffith University, Southport, QLD, Australia; ^8^Molecular Therapy Group, Institute of Biotechnology, Czech Academy of Sciences, Prague, Czechia; ^9^Section on Endocrinology and Genetics, Eunice Kennedy Shriver National Institute of Child Health and Human Development, National Institutes of Health, Bethesda, MD, United States; ^10^Endocrine Oncology Branch, Center for Cancer Research, National Cancer Institute, National Institutes of Health, Bethesda, MD, United States; ^11^Department of Nuclear Medicine, La Timone University Hospital, Aix-Marseille University, Marseille, France; ^12^Department of Pediatrics, RJ and LA Carver College of Medicine, University of Iowa, Iowa City, IA, United States; ^13^Neuroendocrine Tumor Program, Division of Endocrinology and Metabolism, Department of Medicine, Holden Comprehensive Cancer Center, The University of Iowa, Iowa City, IA, United States; ^14^Nuclear Medicine Division, Radiology and Imaging Sciences, Warren Grant Magnuson Clinical Center, National Institutes of Health, Bethesda, MD, United States; ^15^Nuclear Medicine, Radiology and Radiological Science, Johns Hopkins Medicine, Baltimore, MD, United States

**Keywords:** paraganglioma, pheochromocytoma, *SDHA*, ^68^Ga-DOTATATE, PET/CT, ^18^F-FDG, ^123^I-MIBG, ^18^F-FDOPA

## Abstract

**Background:** Pheochromocytoma and paraganglioma (PHEO/PGL) are rare neuroendocrine tumors which may cause potentially life-threatening complications, with about a third of cases found to harbor specific gene mutations. Thus, early diagnosis, treatment, and meticulous monitoring are of utmost importance. Because of low incidence of succinate dehydrogenase complex subunit A (*SDHA*)-related metastatic PHEO/PGL, currently there exists insufficient clinical information, especially with regards to its diagnostic and treatment characteristics.

**Methods:** Ten patients with *SDHA*-related metastatic PHEO/PGL were followed-up prospectively and/or retrospectively between January 2010–July 2018. They underwent biochemical tests (*n* = 10), ^123^I-MIBG (*n* = 9) scintigraphy, and multiple whole-body positron emission tomography/computed tomography (PET/CT) scans with ^68^Ga-DOTATATE (*n* = 10), ^18^F-FDG (*n* = 10), and ^18^F-FDOPA (*n* = 6).

**Results:** Our findings suggest that these tumors can occur early and at extra-adrenal locations, behave aggressively, and have a tendency to develop metastatic disease within a short period of time. None of our patients had a family history of PHEO/PGL, making them appear sporadic. Nine out of 10 patients showed abnormal PHEO/PGL-specific biochemical markers with predominantly noradrenergic and/or dopaminergic phenotype, suggesting their utility in diagnosing and monitoring the disease. Per patient detection rates of ^68^Ga-DOTATATE (*n* = 10/10), ^18^F-FDG (*n* = 10/10), ^18^F-FDOPA (*n* = 5/6) PET/CT, and ^123^I-MIBG (*n* = 7/9) scintigraphy were 100, 100, 83.33, and 77.77%, respectively. Five out of 7 ^123^I-MIBG positive patients had minimal ^123^I-MIBG avidity or detected very few lesions compared to widespread metastatic disease on ^18^F-FDG PET/CT, implying that diagnosis and treatment with ^123/131^I-MIBG is not a good option. ^68^Ga-DOTATATE PET/CT was found to be superior or equal to ^18^F-FDG PET/CT in 7 out of 10 patients and hence, is recommended for evaluation and follow-up of these patients. All 7 out of 7 patients who received conventional therapies (chemotherapy, somatostatin analog therapy, radiation therapy, ^131^I-MIBG, peptide receptor radionuclide therapy) in addition to surgery showed disease progression.

**Conclusion:** In our cohort of patients, *SDHA*-related metastatic PHEO/PGL followed a disease-course similar to that of *SDHB*-related metastatic PHEO/PGL, showing highly aggressive behavior, similar imaging and biochemical phenotypes, and suboptimal response to conventional therapies. Therefore, we recommend careful surveillance of the affected patients and a search for effective therapies.

## Introduction

Pheochromocytomas and paragangliomas (PHEOs/PGLs) are rare neuroendocrine tumors that produce catecholamines which may cause potentially life-threatening complications. Recently, there has been a vast influx of information regarding the genetic basis of these tumors (1–5). Our current understanding is that most of these tumors are inherited (1, 3).

The SDH (succinate dehydrogenase) respiratory complex with a role in the Krebs cycle and oxidative phosphorylation (6) has four subunits encoded by 4 *SDHx* genes-*SDHA, SDHB, SDHC*, and *SDHD* that act as tumor suppressors (7–15). Initially, the *SDHA* gene had been associated only with Leigh syndrome (4, 16). Subsequent studies linked mutations in this gene to abdominal PGL, pituitary adenoma, gastrointestinal stromal tumors (GIST), neuroblastoma, and renal cell carcinoma (RCC) (8, 12, 13, 17–27). *SDHA*-related PHEO/PGL is less well-characterized due to low incidence (17, 28, 29). However, emerging data shows that *SDHA*-related PHEO/PGL is likely to be highly associated with metastases (29). Unfortunately, there is a paucity of data regarding *SDHA*-related metastatic disease. Inadequate knowledge of these tumors poses challenges to those who counsel and treat affected patients (30).

The objective of the present study is to perform a comprehensive review of *SDHA*-related metastatic PHEOs/PGLs and to provide new insights into their clinical behavior, course of disease, biochemical and imaging phenotypes, their possible association with other diseases/tumors, and treatment responses that could help concerned specialists formulate diagnostic and management strategies.

## Patients and Methods

Fifteen patients with PHEO/PGL related to *SDHA* germline mutations were referred to the National Institutes of Health (NIH), a tertiary specialized center for PHEO/PGL. These patients were enrolled and retrospectively and prospectively followed from January 2010–July 2018 under the protocol Diagnosis, Pathophysiology, and Molecular Biology of Pheochromocytoma and Paraganglioma (ClinicalTrials.gov Identifier: NCT00004847), which was approved by the *Eunice Kennedy Shriver* National Institutes of Child Health and Human Development and Institutional Review Board. Written informed consent was obtained from all participants or their legal guardians. Ten of these 15 patients with documented imaging and/or histopathological evidence/s of metastatic disease as per the 2017 World Health Organization classification (lesions in the bone and/or lymph nodes) were included in this study. At their initial evaluation at the NIH, all 10 patients were found to have metastatic disease and 6 out of 10 patients had prior documentation of *SDHA* mutation. Five of the 15 patients without documented metastatic disease were excluded. At our institution, the patients underwent biochemical tests (*n* = 10), [^68^Ga]-(DOTA)-[Tyr3]-octreotate (^68^Ga-DOTATATE, *n* = 10), [^18^F]-fluorodeoxyglucose (^18^F-FDG, *n* = 10), [^18^F]-fluorodihydroxyphenylalanine (^18^F-FDOPA; *n* = 6, patients 2, 4, 6, and 8–10) positron emission tomography/computed tomography (PET/CT), and ^123^I-metaiodobenzylguanidine single photon emission computed tomography/computed tomography (^123^I-MIBG SPECT/CT; *n* = 4, patients 1–3 and 8) scintigraphy. Five patients also received ^123^I-MIBG (planar; *n* = 1, patient 5; SPECT only; *n* = 1, patient 10; and SPECT/CT; *n* = 3, patient 4, 7, and 9) scintigraphy at outside institutions which were included in this report. Pertinent events that happened to the patient prior to referral to our institution were also obtained to review the entire disease course. With respect to the evolution of metastatic lesions, patients were further divided into those with synchronous vs. metachronous disease, defined as the diagnosis of metastases within and beyond 6 months post PHEO/PGL diagnosis, respectively. Table 1 summarizes the patients' main characteristics.

**Table 1:** *SDHA*-related metastatic PHEO/PGL demographic, genetic mutation, and clinical profile.

**Patient number**	**Age at PHEO/PGL detection (in years)/ gender**	**Presented signs and/or symptoms prior to diagnosis**	**Race**	**Primary tumor location and size (in cm)**	**Mutation type**	**Gene locus**	**Effect**	**Location of metastatic lesions**	**Lung or Liver involvement**	**Temporal evolution of metastases; length of time from detection of primary tumor to detection of metastases (in months)**	**Age at metastases detection (in years)**	**Therapy**	**Time to recurrence of resected tumor (in months)**	**Course and outcome**
1	11/F	Left-sided neck mass	Caucasian	Head and neck (6 cm)	Non-sense point mutation	c.91C>T (p.Arg31*)	Pathogenic	Bones and lymph nodes	Lungs	Metachronous; 12 months	12	Surgery	36	Initial recurrence and progression after surgery then disease stability with repeat surgery
2	53/F	Hypertension, abdominal pain, nausea, palpitations, orthostatic light-headedness, and dizziness	Caucasian	Retroperitoneal (more than 6 cm)	Non-sense point mutation	c.1534C>T (p.Arg512*)	Pathogenic	Bones and lymph nodes	Lungs	Synchronous; detected at the same time as the primary tumor	53	Partial resection; somastostatin analog; ^90^Y-DOTATOC; ^177^Lu-DOTATOC; CVD chemotherapy; bortezomib and clofarabine experimental therapy; combination capecitabine and TMZ chemotherapy	3	Residual disease, progression, and death after 26 months of last cycle of PRRT
3	14/M	Hypertension, nausea, fatigue, flushing, palpitation, weight loss	Caucasian	Retroperitoneal (9 cm)	Non-sense point mutation	c.91C>T (p.Arg31*)	Pathogenic	Bones and lymph nodes	No	Synchronous; detected at the same time as the primary tumor	14	Surgery; ^90^Y-DOTATOC; somastostatin analog; ONC201 experimental therapy	2	Initial recurrence and progression after surgery then disease stability for 14 months with ^90^Y-DOTATOC, followed by disease progression and then disease stability with somastostatin analog and ONC201 experimental therapy
4	57/M	Hypertension	Caucasian	Retroperitoneal (4.2 cm); Head and neck (size not specified due to very small size)	Non-sense point mutation	c.91C>T (p.Arg31*)	Pathogenic	Lymph node	No	Metachronous; 7 months	57	Surgery	7	Recurrence and progression followed by disease stability without further therapy
5	53/M	Mediastinal mass, hypertension	Caucasian	Mediastinum (4 cm)	Missense point mutation	c.1334C>T (p.S445L)	Variant of uncertain significance	Bones	No	Metachronous; 48 months	57	Surgery; somatostatin analog; radiosurgery; somatostatin analog and metronomic doses of TMZ; propranolol	38	Initial progression then disease stability with radiosurgery, somatostatin analog and TMZ, however somatostatin analog and TMZ were withdrawn due to side effects, disease stability with propranolol followed by disease progression and resumption of somatostatin analog and metronomic doses of TMZ therapy
6	20/M	Stroke features, hypertension, right flank pain	Caucasian	Retroperitoneal (4.4 cm)	Non-sense point mutation	c.91C>T (p.Arg31*)	Pathogenic	Bones and lymph nodes	No	Synchronous; 2 months	20	Surgery	31	Initial recurrence and progression after surgery then disease stability without further therapy
7	56/M	Hypertension, palpitation, left flank pain	Caucasian	Left adrenal (4.9 cm)	Non-sense point mutation	c.91C>T (p.Arg31*)	Pathogenic	Bones and lymph nodes	Lungs and liver	Metachronous; 120 months	62	Surgery; external radiation therapy; ^131^I-MIBG; CVD chemotherapy	NA	Unknown disease status for 10 years after surgery with rapid progression thereafter despite external radiation, ^131^I-MIBG, and CVD therapies, and death after 20 months of ^131^I-MIBG therapy
8	29/F	Epigastric pain, weakness	Caucasian	Porta hepatis (10.4 cm)	Deletion	c.5′UTR_3′ UTRdel	Pathogenic	Bones and lymph nodes	Lungs	Metachronous; 20 months	30	Surgery; decompression surgery; external radiation therapy; ^177^Lu-DOTATATE	12	Initial recurrence and progression after surgery, then disease stability for 14 months after decompression surgery and external radiation therapy; completed 4 cycles of ^177^Lu-DOTATATE therapy
9	46/F	Abdominal pain	Caucasian	Retroperitoneal (6.8 cm)	Non-sense point mutation	c.91C>T (p.Arg31*)	Pathogenic	Bones and lymph nodes	Lungs and liver	Metachronous; 78 months	53	Surgery; external radiation therapy; hepatic embolization; ^177^Lu-DOTATATE; planned CVD	84	Unknown disease status for 7 years after surgery with rapid progression thereafter; progressed following two cycles of ^177^Lu-DOTATATE therapy
10	44/M	Neck pain	Caucasian	Retroperitoneal (7.7 cm)	Non-sense point mutation	c.91C>T (p.Arg31*)	Pathogenic	Bones and lymph nodes	Lungs	Synchronous; detected at the same time as the primary tumor	44	Cervical spine decompression and fusion surgery; ^131^I-MIBG; chemotherapy with standard dose of TMZ; chemoswitch with metronomic doses of TMZ	8	Progression after surgery and 3 cycles of ^131^I-MIBG treatment, then progression after 3 courses of TMZ

*CVD, combination chemotherapy with cyclophosphamide, vincristine, and dacarbazine; F, female; M, male; NA, not applicable; PHEO, pheochromocytoma; PGL, paraganglioma; SDHA, succinate dehydrogenase complex subunit A; TMZ, temozolomide; ^131^I-MIBG, ^131^I-metaiodobenzylguanidine; ^177^Lu-DOTATOC, [^177^Lu-DOTA]^0^-D-Phe^1^-Tyr^3^-Octreotide; ^90^Y-DOTATOC, [^90^Y-DOTA]^0^-D-Phe^1^-Tyr^3^-Octreotide; PRRT, peptide receptor radionuclide therapy*.

### Patient 1

An 11-year-old female presented with an incidental left-sided neck mass. Computed tomography (CT) scan revealed a 6.0 cm mass that was biopsied and found to be a PGL. At age 12, ^123^I-MIBG SPECT/CT scintigraphy showed lack of tracer avidity of the described mass. She had surgical resection at this age and was found to have a vagal PGL and a solitary lymph node involvement. Monitoring with whole body magnetic resonance imaging (MRI) scan for the next 3 years showed no recurrence or metastases. At the age of 15 however, her CT scan revealed a recurrent 1.0 cm left-sided neck mass, multiple subcentimeter bilateral lung lesions and, 1.0 and 0.8 cm pancreatic body and tail masses, respectively. A ^123^I-MIBG SPECT/CT scintigraphy remained negative. All biochemical tests remained normal. No treatment was initiated. Periodic surveillance for the next 3 years showed stable disease until she was lost to follow-up. Although asymptomatic, progressive disease was found at the age of 21, involving 2 left cervical lymph nodes measuring 2.0 and 0.9 cm, and left 2nd rib and left iliac bone lesions all positive on CT, MRI, ^68^Ga-DOTATATE, and ^18^F-FDG PET/CT scans. She underwent modified radical left neck dissection. Pathology revealed multiple cervical lymph node metastases, with the largest measuring 2.7 cm. One year later, the patient was found to be stable on CT, MRI, and ^68^Ga-DOTATATE PET/CT scans and biochemical tests remained normal. No further treatment was initiated.

### Patient 2

A 53-year-old female presented with metastatic retroperitoneal PGL. Her initial biochemical tests revealed elevated urine norepinephrine (NE), dopamine (DA), and plasma normetanephrine (NMN). CT and MRI scans revealed a 6.0 cm retroperitoneal mass encircling the aorta. Although her ^68^Ga-DOTATATE, ^18^F-FDG, and ^18^F-FDOPA PET/CT scans revealed varied detection rates of multiple bone/bone marrow lesions and a right lung lesion, her ^123^I-MIBG SPECT/CT scintigraphy demonstrated only the retroperitoneal mass.

Due to the invasion of the retroperitoneal mass into the left aortic wall, it could only be partially resected. Histopathology confirmed the presence of PGL measuring 9.0 cm. Short-acting octreotide administered subcutaneously (sc) at 25 micrograms twice daily was initiated post-operatively with no relief of symptoms and little reduction in the level of catecholamines. Three months after surgery, disease progression was noted on MRI scan, with re-demonstration of the partially resected retroperitoneal PGL measuring 3.0 cm. There was an interval appearance of several lymph node and bone metastases. She underwent peptide receptor radionuclide therapy (PRRT) with 160 mCi of [^90^Y-DOTA]^0^-D-Phe^1^-Tyr^3^-Octreotide (^90^Y-DOTATOC) and 2 doses of 200 mCi of [^177^Lu-DOTA]^0^-D-Phe^1^-Tyr^3^-Octreotide (^177^Lu-DOTATOC) over a duration of 5-months. There were significant decreases in chromogranin A (CgA), urine DA, and plasma and urine NMN and NE. Her plasma metanephrine (MN) level was elevated, which was thought to be partly “stress” related. CT and MRI scans showed mixed results: a significant decrease in size of the retroperitoneal PGL from 3.0 cm pre-PRRT therapy to 1.8 cm post- PRRT therapy, decrease in size of the cervical and abdominal lymph nodes, and unchanged thoracic lymph node and lung lesions. There was interval appearance of new metastatic bone lesions and both enlargement and regression of previous bone lesions. ^18^F-FDG PET/CT scan showed a decrease in metabolic activity of the retroperitoneal PGL, cervical, thoracic, and abdominal lymph node lesions. ^18^F-FDG PET/CT scan likewise demonstrated decreased activity and disappearance of some of the bone lesions but increased activity of one bone lesion.

Eight months after her PRRT, she developed progressive disease, elevated CgA, methoxytyramine (MTY), plasma DA, plasma, and urine NMN and NE and extensive bone metastases on imaging. CT, MRI, and ^18^F-FDG PET/CT scans revealed a residual retroperitoneal mass, stable cervical lymph node lesions, increase in the size of one of the abdominal lymph node lesions, and both interval progression and development of new bone lesions. Chemotherapy with cyclophosphamide, vincristine, and dacarbazine (CVD) was started and resulted in a mixed response with a decrease in the size and activity of the abdominal lymph node lesions, stability in the size of the thoracic lymph node and lung lesions, and both stability and increase in the number and size of bone lesions as demonstrated by CT, MRI, and ^18^F-FDG PET/CT scans. Chemotherapy was discontinued after 10 cycles due to pancytopenia, which could have been caused by PRRT. She then had further disease progression. Bortezomib and clofarabine experimental therapy was subsequently tried but had further disease progression and hence, patient was started on combination capecitabine and temozolomide. However, the patient expired the following year.

### Patient 3

A 14-year-old male was diagnosed with metastatic abdominal PGL. Biochemical tests at that time showed elevated CgA, urine NMN and NE. CT and MRI scans showed an abdominal PGL measuring 9.1 cm with invasion of the inferior vena cava (IVC) and bone metastases. Resection of the abdominal PGL was performed. Histopathology revealed a PGL measuring 9.0 cm. Two months after surgery, CT angiography revealed a 2.5 cm recurrent abdominal mass. ^18^F-FDG PET/CT scan confirmed this mass with several additional bone metastases. ^123^I-MIBG SPECT/CT scintigraphy did not show avidity for the aforementioned lesions.

One year later, MRI scan showed an interval increase in the size of the abdominal PGL to 3.0 cm, a thoracic soft tissue lesion, and multiple lymph node and bone metastases. ^18^F-FDG PET/CT scan demonstrated recurrent abdominal PGL and bone metastases. Only close monitoring was done due to the slow disease progression, limited chemotherapeutic options, and absence of alarming symptoms.

Four months later, MRI, ^68^Ga-DOTATATE, and ^18^F-FDG PET/CT scans again demonstrated slowly progressive metastatic disease. He received 3 cycles of PRRT with 100 mCi each of ^90^Y-DOTATOC. Eight months later, CT and [^68^Ga-DOTA]^0^-D-Phe^1^-Tyr^3^-Octreotide (^68^Ga-DOTATOC) PET/CT scans showed stable disease. However, 6 months later, the CT scan demonstrated an interval increase in size of the abdominal PGL to 4.3 cm without any evidence of new lesions on ^18^F-FDG PET/CT scan. Hence, he was started on lanreotide 120 mg/sc every 28 days and experimental therapy with ONC201 625 mg/weekly and demonstrated stable disease on ^18^F-FDG PET/CT and CT scan after 3 and 6 months, respectively, along with a decrease in plasma CgA at both time points.

### Patient 4

A 57-year-old male was diagnosed with metastatic para-aortic abdominal PGL. During work-up for hypertension, CT scan revealed a 5.1 cm retroperitoneal para-aortic mass, a 2.5 cm right renal superior pole mass, and a 2.5 cm left adrenal mass, which was later found to be a non-functioning adenoma. The ^123^I-MIBG SPECT/CT scintigraphy was positive only for the para-aortic mass. ^18^F-FDG PET/CT scan showed avidity for the para-aortic mass and mild uptake for the gastric cardia. Initial biochemical tests were normal. Resection of the para-aortic and gastric lesion masses, and right partial nephrectomy were performed. Histopathology confirmed a 4.2 cm para-aortic PGL, RCC, and GIST. Immunohistochemical staining (IHC) for GIST demonstrated loss of SDHB staining without loss of SDHA staining, whereas RCC demonstrated loss of neither SDHB nor SDHA staining.

Seven months later, ^18^F-FDOPA and ^68^Ga-DOTATATE PET/CT showed recurrence of the retroperitoneal PGL with a subcentimetric soft tissue lesion in the left neck, considered another primary head and neck PGL, which along with mediastinal lymph node metastasis could not be localized by neck MRI scan retrospectively.

Two years later, MRI scan showed stable left adrenal mass, vertebral hemangiomas, liver and renal cysts, elevated plasma epinephrine (EPI) and DA, and significant uptake on ^68^Ga-DOTATATE and ^18^F-FDOPA PET/CT on the post-operative site, cervical, and thoracic area. No treatment was initiated.

Two years later, MRI, ^68^Ga-DOTATATE, and ^18^F-FDG PET/CT scans demonstrated stable disease and no treatment was initiated.

### Patient 5

A 53-year-old male presented with a 4.0 cm right posterior mediastinal mass which was incidentally discovered on his chest radiograph, and subsequently by ^18^F-FDG PET/CT scan. Urine NMN and NE were elevated. Excision of the mass was done, and histopathology confirmed the PGL.

Three years and 2 months later, he showed recurrence of the mediastinal mass on MRI scan and a year and a half later, he developed painful bone metastases which were minimally avid on ^123^I-MIBG planar scintigraphy. ^111^In-pentetreotide scintigraphy had findings suggestive of metastatic disease. At this time, urine MN and CgA were elevated. He was started on lanreotide 120 mg/sc every 14 days which resulted in pain control, stabilization of bone metastases, and reduction and normalization of urine MN and CgA, respectively. This was followed by radiation therapy to bone metastases. Seven months later, the lanreotide dose was tapered to 120 mg/sc every 21 days due to development of side effects and antihypertensive management was switched from bisoprolol to propranolol 120 mg/day.

A year and a half later, the patient developed progressive bone metastases. He was started on metronomic doses of temozolomide (TMZ) 75 mg/m^2^ for 21 days every 4 weeks in addition to lanreotide therapy and the dose of propranolol was increased to 240 mg/day resulting in stable disease 3 months later on CT, MRI, ^18^F-FDG, and ^68^Ga-DOTATATE PET/CT scans. At that time, CgA, plasma DA, urine NE, and urine NMN were elevated. Five months later,^18^F-FDG PET/CT scan showed stable disease and at that time lanreotide was withdrawn due to development of nausea, vomiting, diarrhea, and hyperglycemia. Repeat ^18^F-FDG PET/CT scan 6 months later showed stable disease and subsequently, TMZ was withdrawn due to long standing grade 2 lymphopenia and the patient continued only on propranolol 240 mg. However, 12 months later, he developed progressive disease on ^18^F-FDG PET/CT scan and a combination of metronomic doses of TMZ (75 mg/m^2^ for 21 days every 4 weeks) and lanreotide 120 mg/sc every 21 days was resumed.

### Patient 6

A 20-year-old male was diagnosed with metastatic retroperitoneal PGL. Biochemical testing showed elevated urine NMN. CT and ^18^F-FDG PET/CT scans showed a retroperitoneal mass anterior to the IVC and a periaortic lymph node metastasis. The retroperitoneal mass on biopsy revealed PGL. Two months later, the periaortic lymph node and retroperitoneal PGL were excised. Histopathology confirmed a 4.4 cm PGL and one metastatic lymph node. ^18^F-FDOPA and ^18^F-FDG PET/CT scans performed 7 months after surgery revealed a right acetabulum metastatic lesion with normal biochemical tests.

Two years and 7 months after surgery, a recurrent retroperitoneal lesion with metastatic lymph nodes and other soft tissues were seen on ^18^F-FDG and ^18^F-FDOPA PET/CT scans in addition to the acetabular lesion. ^68^Ga-DOTATATE PET/CT scan was positive only for the acetabular lesion. Biochemical tests were normal. One year later, ^18^F-FDG, ^68^Ga-DOTATATE, and ^18^F-FDOPA PET/CT scans and biochemical tests showed stable disease. To date, no treatment for metastatic disease has been initiated.

### Patient 7

A 52-year-old male initially presented with hypertension and palpitations. Four years later, during work-up for left flank pain, a large retroperitoneal tumor was incidentally discovered. Presumably arising from the kidney, he underwent left nephrectomy with left adrenalectomy. Histopathology confirmed PHEO in the resected 4.9 cm retroperitoneal mass and in a metastatic lymph node, however, there was no renal involvement.

Ten years after the surgery, a left upper back mass was discovered. MRI scan revealed a 5.0 cm T3 vertebral body lesion extending into the epidural space with resultant cord compression. ^18^F-FDG PET/CT scan demonstrated metastatic bone disease involving C2-C3, T3, L5, and right iliac bone. Biochemical testing revealed elevated urine NE. He underwent T3 spinal tumor resection. Histopathology confirmed a 5.7 cm PGL with positive margins. Two months later, the patient received radiation therapy with a total of 30 gray divided into 10 fractions along C6 through T5. At that time, ^123^I-MIBG SPECT/CT scintigraphy showed uptake in T3 and S1 bone lesions.

Three months after external radiation therapy, he received 319 mCi of ^131^I-MIBG therapy. Post-therapy whole-body ^131^I-MIBG SPECT/CT scintigraphy showed uptake in the thoracic vertebrae and other axial bone lesions with a new bone lesion in the right femur. Three months after ^131^I-MIBG therapy, he underwent shave biopsy of new skin lesions in left lower shin and right-hand dorsum, which were found to be well-differentiated squamous cell carcinoma.

After 6 months of ^131^I-MIBG therapy, the ^123^I-MIBG SPECT/CT scintigraphy showed decreased uptake in the index lesions at T3 and S1 as compared to pre-therapy ^123^I-MIBG SPECT/CT scintigraphy without any new foci of increased radiotracer uptake from post-therapy ^131^I-MIBG SPECT/CT scintigraphy. However, ^123^I-MIBG SPECT/CT scintigraphy after 12 months of ^131^I-MIBG therapy revealed disease progression with extensive metastatic bone disease involving most of the spine, pelvis, and patchy areas of uptake in the scapulae, proximal humeri, proximal femurs, sternum, and multiple bilateral ribs. A year after initiation of ^131^I-MIBG therapy, metastatic disease involving the bones, bone marrow, lungs, and liver were found on ^68^Ga-DOTATATE and ^18^F-FDG PET/CT. During this time, plasma, and urine DA, NE, and NMN, and plasma MN and MTY were elevated. CVD chemotherapy was started on a 21-day cycle. The patient progressed 4 months later on ^18^F-FDG PET/CT with appearance of new lesions in the lungs. He was evaluated for pembrolizumab experimental therapy, however, he expired 4 months later due to suspected leptomeningeal disease.

### Patient 8

A 29-year-old female initially presented with epigastric pain and weakness. CT scan showed a large 10.4 cm vascular mass in the porta hepatis, possibly obliterating the right adrenal gland, thought to be an arteriovenous malformation for which she received 4 cycles of coil embolization therapy. Subsequently, she developed lower limb weakness and difficulty in walking. A year and a half later, MRI spine showed a T7 bone lesion with extradural extension and soft tissue swelling with severe spinal cord compression, which was treated with endovascular embolization. However, 2 months later, due to worsening symptoms, she underwent vertebrae body stabilization and excision of the tumor, which was confirmed to be a metastatic PGL on histopathology. Biochemical testing revealed elevated plasma NE and CgA.

Within a year, she again developed high-grade spinal cord compression from a recurrent T7 vertebral body bone lesion, which was visible on ^68^Ga-DOTATOC PET/CT along with lesions in the skull base, cervical spine, right first rib, right iliac bone, both lungs, and the right adrenal bed. She underwent emergent surgical decompression of the T7 lesion and 1 month later received post-operative radiation of 54 grays divided over 30 fractions from T5 through T9. She remained stable without any disease progression for the following 14 months.

**Figure 1:**
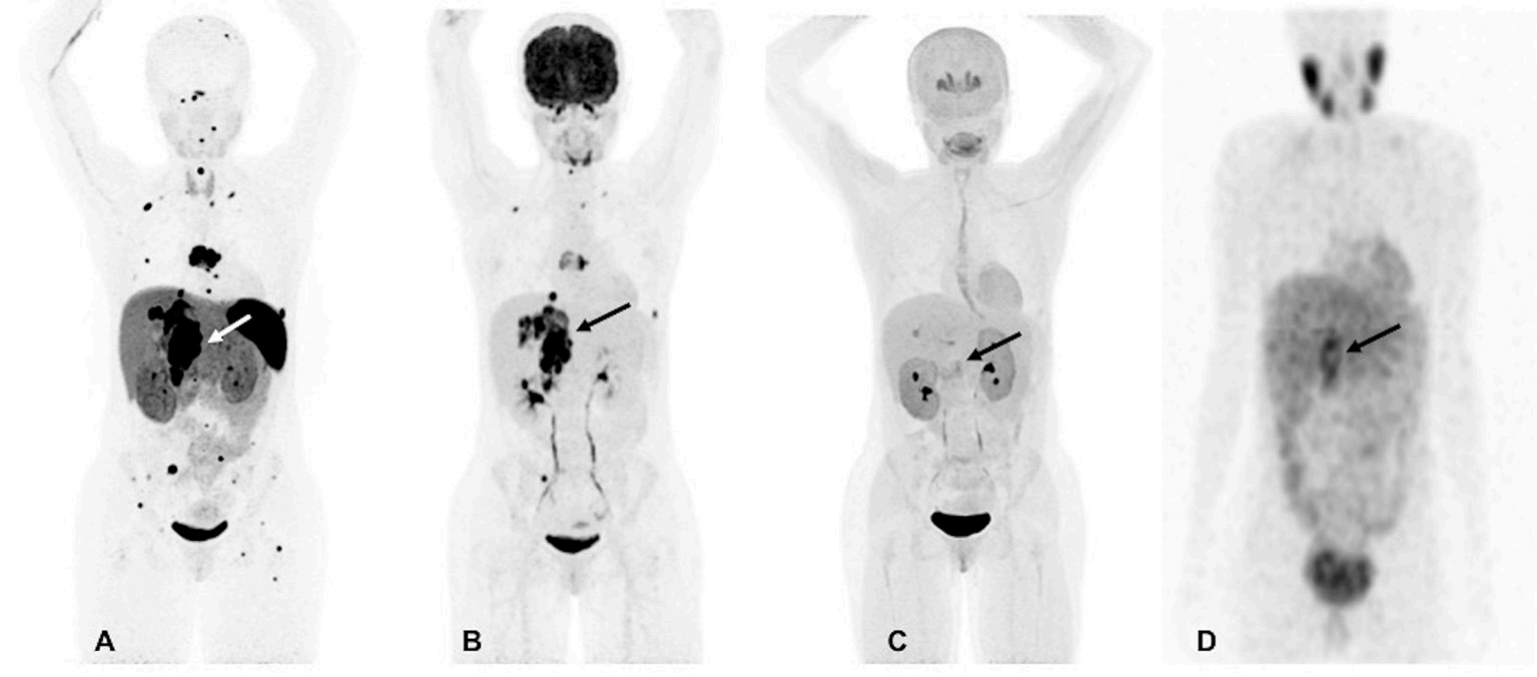
Functional imaging in a *SDHA*-related metastatic PHEO/PGL. In this figure of a 33-year-old female patient (patient 8) demonstrating the superiority of ^68^Ga-DOTATATE PET/CT in comparison to other modalities, anterior maximum intensity projection (MIP) images of ^68^Ga-DOTATATE PET/CT **(A)**, ^18^F-FDG PET/CT **(B)**, ^18^F-FDOPA PET/CT **(C)**, and anterior planar view whole body image of ^123^I-MIBG scintigraphy **(D)** scans demonstrate a large retroperitoneal tumor (arrows). The ^68^Ga-DOTATATE **(A)** and ^18^F-FDG PET/CT **(B)** scans show widespread metastases in bone and lymph nodes along with lesions in both lungs. The ^68^Ga-DOTATATE PET/CT scan detects more bone lesions than ^18^F-FDG PET/CT scan with a higher tumor uptake making the lesions on ^68^Ga-DOTATATE PET/CT scan relatively more conspicuous than ^18^F-FDG PET/CT scan. The duration between ^68^Ga-DOTATATE PET/CT scan and ^18^F-FDG PET/CT, ^18^F-FDOPA PET/CT, and ^123^I-MIBG scintigraphy were 1 day, 3 days, and 2 months, respectively.

However, at 33, there was again recurrence in the T7 vertebral body and the lesion in the right adrenal bed, along with scattered metastatic bone and lung lesions which were visible on both ^18^F-FDG and ^68^Ga-DOTATATE PET/CT scans. The ^18^F-FDOPA and ^123^I-MIBG SPECT/CT scans demonstrated a much lower number of metastatic lesions and were found to be inferior in comparison to ^68^Ga-DOTATATE PET/CT (Figure 1). At that time, her plasma NMN and CgA were elevated. Surgical intervention was deemed high-risk, therefore, she received four cycles of PRRT treatment with 200 mCi of ^177^Lu-DOTATATE without any adverse events and was found to be stable on CT, ^18^F-FDG, and ^68^Ga-DOTATATE PET/CT scans 1-month after completion of PRRT. At that time, there was significant decrease in her plasma NMN and CgA.

### Patient 9

A 46-year-old woman presented with abdominal pain and her CT scan revealed a large retroperitoneal tumor, which was surgically resected and found to be a 6.8 cm PGL on histopathology. She remained disease free for the following 6 years.

At age 53, the patient reported left shoulder pain which on MRI scan revealed a lytic lesion in the coracoid process and received 2.5 gray of external beam radiotherapy over 13 fractions. Subsequent CT and ^68^Ga-DOTATATE PET/CT scans additionally showed liver lesions ranging from 3.9 to 5.9 cm in size, bilateral lung lesions, multiple metastatic neck, mediastinal, retroperitoneal lymph nodes, and a left inferior pubic ramus bone metastasis. However, on ^123^I-MIBG SPECT/CT scintigraphy, only the left coracoid process and left inferior pubic ramus bone metastases were visible. An ultrasound guided liver biopsy was performed, and pathology revealed a metastatic PGL. Biochemical tests revealed elevated plasma NMN, MTY, and CgA. A follow up CT scan after 2 months revealed an interval increase in the size of the liver lesions and recurrence of the retroperitoneal PGL. Subsequent imaging with ^68^Ga-DOTATATE, ^18^F-FDG, and ^18^F-FDOPA PET/CT scans re-demonstrated the aforementioned lesions. One of the liver lesions located in the left hepatic lobe did not show any DOTATATE avidity so she underwent embolization of this lesion in anticipation of starting PRRT with ^177^Lu-DOTATATE. She subsequently received external beam radiotherapy to the pubic bone then underwent 2 of the 4 cycles of ^177^Lu-DOTATATE therapy without any complications. However, she showed progression on CT, ^18^F-FDG, and ^68^Ga-DOTATATE PET/CT scans and hence, ^177^Lu-DOTATATE therapy was stopped. At that time, her plasma CgA and MTY were elevated and had increased in comparison to pre-PRRT levels, whereas plasma NMN had normalized. Following the progression of her disease, she was recommended to undergo CVD chemotherapy.

### Patient 10

**Figure 2:**
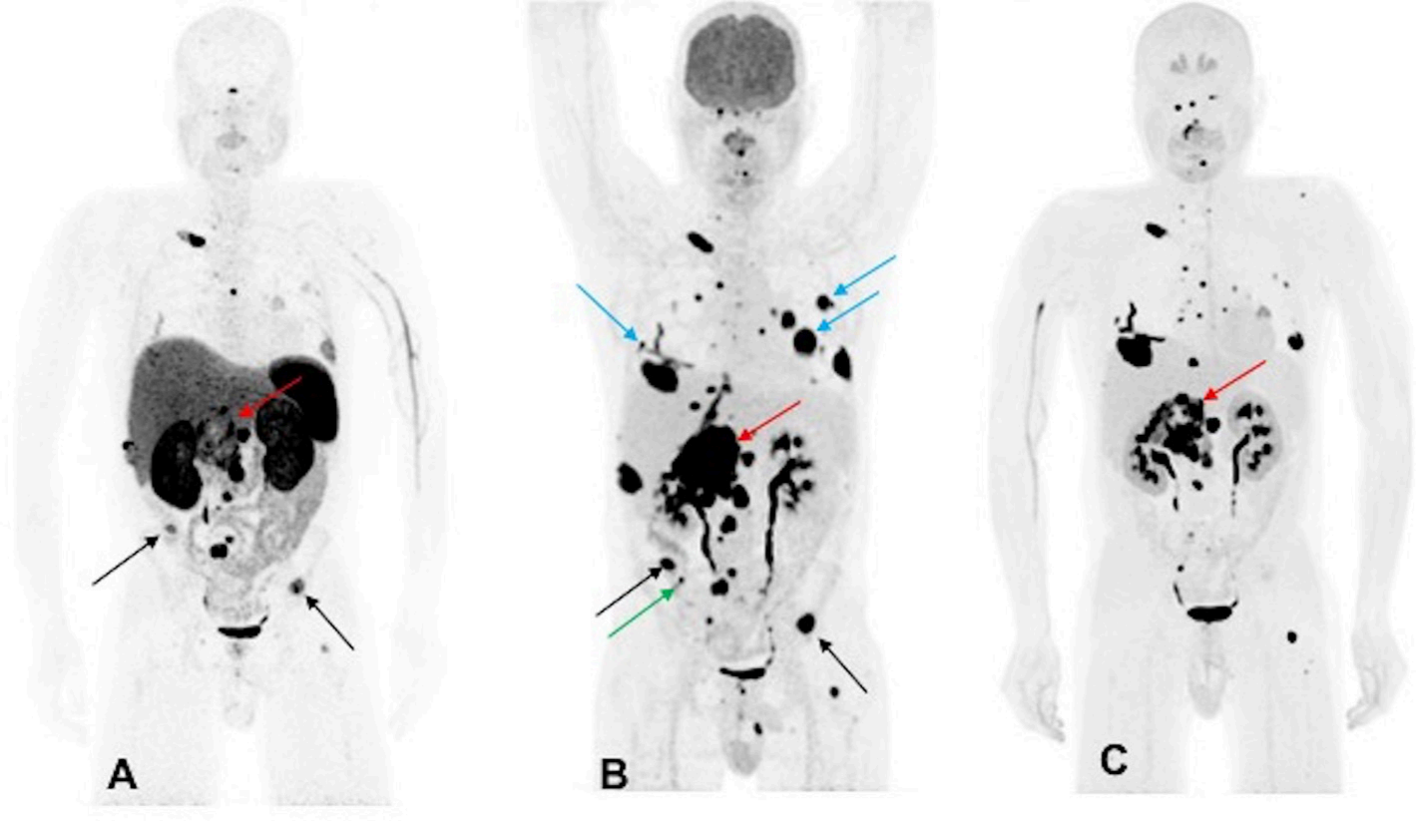
Functional imaging in a *SDHA*-related metastatic PHEO/PGL. In this figure of a 45-year-old male patient (patient 10) demonstrating the inferiority of ^68^Ga-DOTATATE PET/CT in comparison to other PET radiotracers, anterior MIP images of ^68^Ga-DOTATATE PET/CT **(A)**, ^18^F-FDG PET/CT **(B)**, and ^18^F-FDOPA PET/CT **(C)** scans demonstrate a large retroperitoneal tumor (red arrows) with less prominent increased uptake on ^68^Ga-DOTATATE PET/CT and widespread metastatic lesions involving multiple bones, lymph nodes, and bilateral lungs. The ^18^F-FDG PET/CT scan detects more lesions in bilateral lungs (blue arrows) and right iliac bone (green arrow) than ^18^F-FDOPA and ^68^Ga-DOTATATE PET/CT scans. Few lesions, such as bilateral iliac bones (black arrows), are demonstrated in both ^18^F-FDG and ^68^Ga-DOTATATE PET/CT scans and not in ^18^F-FDOPA PET/CT scan. In general, most lesions on ^68^Ga-DOTATATE PET/CT scan show a poor tumor uptake making the lesions on ^68^Ga-DOTATATE PET/CT scan relatively inconspicuous compared with ^18^F-FDG and ^18^F-FDOPA PET/CT scans. The duration between ^68^Ga-DOTATATE PET/CT scan and ^18^F-FDG PET/CT, and ^18^F-FDOPA PET/CT, were 1 and 2 days, respectively.

A 44-year-old male developed neck pain and was subsequently found to have a 3.0 cm C2 vertebral body lesion on CT spine. He developed new onset dysphagia and further imaging work up revealed a lesion anterior to C5-T1, which appeared to be displacing the esophagus along with a heterogenous 7.7 cm retroperitoneal tumor in the right peri-adrenal region which was biopsied and revealed PGL on histopathology. The scan also revealed a right fifth rib, T11, S1, and bilateral lung metastases. The biochemistry tests showed slight elevation of urine NMN, DA, and EPI as well as plasma NE and CgA. He underwent ^123^I-MIBG SPECT scintigraphy 1 month later which revealed uptake in C2 lesion, right anterior and inferior lateral rib cage, anterior first rib, S1, and retroperitoneal tumor. The patient underwent cervical spine decompression and fusion surgery, with subsequent radiation to the cervical spine, and then received three cycles of ^131^I-MIBG treatment. The 6-month post-MIBG therapy restaging ^123^I-MIBG SPECT scintigraphy demonstrated disease progression. The CT scan showed an increase in the number of lung lesions and an increase in the size of the retroperitoneal mass and retroperitoneal lymph nodes. A tumor vs. bland thrombus was also observed in the IVC and the patient was put on anticoagulant therapy. He was started on TMZ (150–200 mg/m^2^ for 5 days every 4 weeks) chemotherapy. Three months later, following 2 cycles of TMZ, the patient progressed per imaging on CT, ^68^Ga-DOTATATE, ^18^F-FDG, and ^18^F-FDOPA PET/CT scans, revealing an increase in size of the retroperitoneal tumor along with bilateral lung lesions and extensive bone metastases (Figure 2). Biochemical tests at that time were normal except for elevated plasma CgA. For treatment of his metastatic disease, the patient was recommended a chemoswitch from standard to metronomic doses of TMZ (75 mg/m^2^ for 21 days every 4 weeks) and is awaiting follow up evaluation.

## Results

Over an 8.5-year period, the proportion of metastatic *SDHA*-related PHEOs/PGLs seen at our institution from all *SDHA* patients was 66.67% (*n* = 10/15). Table 1 shows that all were Caucasian (*n* = 10/10) and 6 out of 10 were males. The median age at disease detection was 45 (range, 11–57) years old. Three out of 10 patients were young at disease onset, diagnosed during the second decade of their lives. Most patients presented with hypertension (*n* = 6/10) followed by abdominal pain (*n* = 5/10), and palpitations (*n* = 3/10). Primary tumors were most commonly located at extra-adrenal sites (*n* = 9/10), particularly in the abdomen and/or retroperitoneum (*n* = 7/10), and less frequently in the neck (*n* = 2/10) and mediastinum (*n* = 1/10). Only one out of 10 patients had a primary tumor located in the left adrenal gland. One out of 10 patients had 2 different primary tumors which were first noted in the retroperitoneum and then in the neck area. The median size of the primary tumors was 6.0 cm (range, 4.0–10.4 cm). Four out of 10 patients had synchronous disease while 6 out of 10 patients had metachronous disease. The median duration of time from the diagnosis of primary tumor to the detection of metastases was 9.5 (range, 0–120) months while the median age for detection of metastases was 48.5 (range, 12–62) years. Bone was the most common metastatic site (*n* = 9/10) followed by lymph nodes (*n* = 8/10). Involvement of lungs was seen in 6 and liver in 2 out of 10 patients. Germline non-sense point mutation in the *SDHA* gene was found to be most predominant, seen in 8 out of 10 patients, mutation with pathogenic effect in 9 out of 10 patients, and mutation with variant of unknown significance in 1 out of 10 patients. The most predominant gene locus for the *SDHA* mutation was noted to be c.91C>T (p.Arg31^*^) in seven out of 10 patients.

Table 2 shows the list of patient- and family-associated neoplasms and autoimmune diseases. Eight out of 10 patients presented with tumors other than PHEO/PGL. Only one patient (patient 4) had IHC of other tumors and demonstrated loss of SDHB staining without loss of SDHA staining for GIST, whereas RCC demonstrated loss of neither SDHB nor SDHA staining. Two out of 10 patients had Hashimoto's thyroiditis. Surprisingly, none presented with a family history of PHEO/PGL. Other tumors (*n* = 8/10) and autoimmune disorders (*n* = 2/10) were present in the family of some. Lung cancer (*n* = 4/10) was the most commonly found cancer in the family. First degree relatives of only 6 patients were tested and all of them were found to carry an *SDHA* mutation (*n* = 6/6). Three of these 6 relatives (*n* = 3/6) were screened for PHEO/PGL by imaging scans and were found to be negative.

**Table 2:** *SDHA* mutation positivity, neoplasms and autoimmune diseases present in the family and patient.

**Patient number**	**Family history of PHEO/PGL**	**Relative/s with *SDHA* mutation**	**Coexisting neoplasms in the patient**	**Family history of neoplasms other than PHEO/PGL**	**Coexisting autoimmune disease in the patient**	**Family history of autoimmune disease**
1	None	Mother^a^; 2 siblings^a^	Pancreatic masses of unknown nature	Small cell lung cancer (maternal uncle)	None	None
2	None	No data	Breast masses of unknown nature, probable vertebral body hemangioma, liver hemangiomas, renal masses possibly cysts, uterine fibroids, submandibular gland cyst	Pancreatic cancer (mother); breast cancer (maternal uncle); glioblastoma (maternal first cousin); melanoma (father); colon cancer (2 paternal cousins); liver cancer (paternal cousin)	Hashimoto's thyroiditis	None
3	None	Father^b^; brother^a^	Liver and lung masses of unknown nature	Lipoma (mother); basal skin cancer (father); prostate cancer (paternal grandfather); lung cancer (paternal grandmother); stomach cancer (paternal grandmother)	None	Rheumatoid arthritis (mother); multiple sclerosis (maternal grandmother)
4	None	No data	GIST, non-functioning left adrenal adenomas, right RCC, esophageal leiomyomas, thyroid cyst, benign lung nodule, liver and renal cysts, vertebral body hemangioma	None	None	None
5	None	No data	Renal cysts	Small cell lung cancer (father)	None	None
6	None	Mother^a^	None	None	None	Sjogren's disease (mother)
7	None	Daughter^a^	Squamous cell carcinoma of skin	Colon cancer (maternal aunt)	None	None
8	None	Sister^b^	Prolactinoma	Uterine or possible ovarian cancer (maternal aunt); prostate cancer (maternal uncle); intestinal cancer (paternal grandmother)	Hashimoto's thyroiditis	None
9	None	Father^b^	None	Breast cancer (sister)	None	None
10	None	No data	Melanoma	Lung cancer (maternal grandmother)	None	None

a *No data on whether biochemical and imaging tests were performed or not*.

b *Had negative imaging finding for PHEO/PGL and no data on whether biochemical tests were performed or not*.

*GIST, gastrointestinal stromal tumor; PHEO, pheochromocytoma; PGL, paraganglioma; RCC, renal cell carcinoma; SDHA, succinate dehydrogenase complex subunit*.

Table 3 shows that elevation in the levels of plasma and/or urine biochemical markers was found in 9 out of 10 patients and levels of NE and/or its metabolite NMN as well as levels of DA and/or its metabolite MTY were elevated in 9 out of 10 patients, whereas EPI and/or its metabolite MN were elevated in 5 out of 10 patients. In contrast, CgA levels were elevated in only 6 out of 9 patients despite the presence of metastatic disease in all patients. Only patient 1 presented with normal biochemical markers despite having metastatic disease.

**Table 3:** Biochemical phenotype of each patient.

**Patient number**	**Plasma**							**Urine**				
	**EPI**	**NE**	**DA**	**MN**	**NMN**	**MTY**	**CgA**	**EPI**	**NE**	**DA**	**MN**	**NMN**
1	–	–	–	–	–	–	–	–	–	–	–	–
2	–	+	+	+	+	+	+	–	+	+	–	+
3	–	–	+	–	–	+	+	–	+	–	–	+
4	+	–	+	–	–	+	–	–	–	–	–	–
5	–	–	+	–	–	–	+	–	+	–	+	+
6	–	–	–	–	–	–	–	–	–	–	–	+
7	–	+	+	+	+	+	*	–	+	+	–	+
8	–	–	–	–	+	–	+	–	–	–	–	–
9	+	–	–	–	+	+	+	–	–	–	–	–
10	–	–	–	–	–	+	+	–	–	–	–	–

*CgA, chromogranin A; DA, dopamine; EPI, epinephrine; MN, metanephrine; MTY, methoxytyramine; NE, norepinephrine; NMN, normetanephrine; +, above upper reference limit; -, within normal limits*.

*,not available due to hemolysis of blood.

All patients underwent functional imaging with ^68^Ga-DOTATATE and ^18^F-FDG PET/CT scans, while 6 patients also underwent ^18^F-FDOPA PET/CT scanning (Supplementary Table 1). The mean duration between ^68^Ga-DOTATATE, ^18^F-FDG, and ^18^F-FDOPA PET/CT scans was 6 days each. All 10 out of 10 patients were positive on ^68^Ga-DOTATATE and ^18^F-FDG PET/CT scans (per patient detection rate of 100%), whereas only 5 out of 6 patients were positive on ^18^F-FDOPA PET/CT (per patient detection rate of 83.33%) scan but with varied per lesion detection rates. In 5 of 10 patients, ^68^Ga-DOTATATE PET/CT scan performed superiorly to ^18^F-FDG PET/CT scan (Figure 1), while in 2 out of 10 patients it performed equally and detected the same number of lesions; however, in 3 out of 10 patients, ^68^Ga-DOTATATE PET/CT scan was inferior to ^18^F-FDG PET/CT scan with respect to number of lesions detected. So, overall ^68^Ga-DOTATATE PET/CT performed superiorly or equally to ^18^F-FDG PET/CT in 7 out of 10 patients. Compared to the ^18^F-FDG PET/CT scan, the performance of ^18^F-FDOPA PET/CT scan was superior and equal in 1 out of 6 patients each, and inferior in 4 out of 6 patients; and overall ^18^F-FDOPA PET/CT scan performed superiorly or equally to ^18^F-FDG PET/CT scan in 2 out of 6 patients. In contrast, most lesions, including metastases, were not ^123^I-MIBG-avid. Nine out of 10 patients who underwent ^123^I-MIBG scintigraphy also underwent ^18^F-FDG PET/CT scan at the same time. The mean duration between ^18^F-FDG PET/CT scan and ^123^I-MIBG scintigraphy study was 2 months with only patient 1 receiving these studies 6 months apart. Nine patients were ^18^F-FDG PET/CT avid with demonstration of widespread metastatic disease in 6 of these 9 patients. ^123^I-MIBG scintigraphy was positive in 7 out of 9 patients (per patient detection rate of 77.77%) whereas 2 out of 9 patients were ^123^I-MIBG-negative, and in 1 of these 2 ^123^I-MIBG-negative patients, widespread metastasis was present. Five out of 7 ^123^I-MIBG positive patients had minimal ^123^I-MIBG avidity or detected very few lesions compared to widespread metastatic disease on ^18^F-FDG PET/CT scan in all 7 patients.

All 10 patients underwent surgical resection of their primary PHEO/PGL or metastases (Table 1). Nine out of 10 patients had recurrent disease with median time to recurrence being 12 (range, 2–84) months. Seven out of 10 patients also received conventional therapy (e.g., chemotherapy, somatostatin analog therapy, radiation therapy, ^131^I-MIBG therapy, peptide receptor radionuclide therapy) apart from surgery and all 7 of them had disease progression despite additional therapy. Mortality was documented in 2 out of 10 patients (patients 2 and 7).

Patient 2 had disease progression despite receiving a somatostatin analog after primary PGL resection and initial mixed response after PRRT followed by rapid progression and eventual death despite trial with various chemotherapeutic agents including experimental chemotherapy. Patients 7 and 10 had rapid disease progression after external radiation therapy and ^131^I-MIBG therapy and hence, the former was started on CVD chemotherapy whereas the latter was started on metronomic doses of temozolomide. Patient 5 had good clinical, biochemical, radiologic response to somatostatin analog therapy followed by radiation therapy, however, had progression after 1.5 years. But metronomic doses of TMZ and lanreotide resulted in radiologically stable disease despite elevated biochemical tests. However, TMZ and lanreotide had to be withdrawn due to side effects and the patient remained stable for a year only on propranolol, but the TMZ and lanreotide had to be reintroduced due to disease progression. Patient 9 remained disease free after surgery for 6 years, however later progressed rapidly despite trial with two cycles of ^177^Lu-DOTATATE therapy whereas patient 8 progressed following surgery and radiation therapy and achieved stability following ^177^Lu-DOTATATE therapy. Patient 3 showed disease stability after ^90^Y-DOTATOC therapy, however progressed later, and hence, started on lanreotide and experimental ONC201 therapy. Patient 4 has achieved disease stability following surgery without additional treatment for 4.5 years now. On the other hand, patients 1 and 6 had initial disease progression and recurrence after surgery followed by disease stability without additional treatment.

## Discussion

In this report, we presented a comprehensive review of the highest recorded number of metastatic *SDHA* patients at one institution and attempted to provide new insights into their clinical behavior, course of disease, biochemical and imaging phenotypes, their possible association with other diseases/tumors, and treatment responses. Other investigations on *SDHA*-related PHEO/PGL (12, 29, 31–35) report the proportion of metastatic disease to be ranging from 9 to 33% (Table 4) compared to 66.67% in our cohort of patients. The proportion of metastatic disease is found to be 20.56% (*n* = 22/107) if all the metastatic patients from reported series (12, 29, 31–35) are pooled with this report.

**Table 4:** Studies showing data of patients with *SDHA*-related metastatic PHEO/PGL.

**Investigators, Year**	**Number with metastatic disease, percentage**	**Ethnicity/ race**	**Patient number assigned**	**Age at PHEO/ PGL detection (in years)/ gender**	**Primary tumor location and size (in cm)**	**Time to recurrence of resected primary tumor (in months)**	**Age at metastases detection (in years)**	**Length of time from detection of primary tumor to detection of metastases (in months)**	**Location of metastatic lesions**	**Mutation type**	**Gene locus**	**Effect**	**Biochemical phenotype**	**Functional imaging**	**Coexisting neoplasms or autoimmune diseases in the patient**	**Family history**	**Therapy**	**Course and outcome**
Korpershoek et al. (12)	1 out of 7, 14.28%	Not reported	Patient 146	41/M	Bladder	Not reported	Not reported	Not reported	Lymph node	Not reported	Not reported	Not reported	NE, E	Not reported	Not reported	Not reported	Not reported	Not reported
Papathomas et al. (34)	1 out of 4, 25%	Not reported	Patient 182	23/M	Not reported	Not reported	Not reported	Not reported	Lymph node	Nonsense mutation	c.1534C>T (p.Arg512*)	Pathogenic	Not reported	Not reported	Not reported	Not reported	Not reported	Not reported
^a^Bausch et al. (31)	4 out of 34, 12%	United States	NA	28/M	Adrenal	Not reported	Not reported	Not reported	Not reported	Not reported	c.1361C>A	Likely pathogenic	Not reported	Not reported	Not reported	Negative	Not reported	Not reported
Casey et al. (32)	1 out of 11, 9.09%	Not reported	NA	43/F	Thoracic	Not reported	Not reported	Not reported	Not reported	Missense mutation	c.923C>T (p.Thr308Met)	^c^Variant of unknown significance	Not specific but reported to be secretory	Not reported	Not reported	Not reported	Not reported	Not reported
^b^Casey et al. (33)	NA	Not reported	NA	23/M	Retroperitoneum	Not reported	46	276	Bone	Truncating mutation	c.91C>T (p.Arg31*)	Pathogenic	Plasma and urine NMN, urine MN	^131^I-MIBG non-avid and ^18^F-FDG PET/CT avid bone lesion	Not reported	Negative	Surgery of primary tumors and metastasis	No new metastasis after surgery of bone lesion
Tufton et al. (29)	2 out of 6, 33.33%	Not reported	Patient 8	46/M	Abdomen (5 cm)	12	63	192	Bone	Missense point mutation	c.923C>T exon 8	^d^Pathogenic	Plasma and urine NE	^18^F-FDG PET/CT avid bone lesions	Negative	No data	Surgery, external beam radiation therapy, radiosurgery	Recurrence after surgery with stability after radiation therapy
			Patient 9	18/F	Right adrenal	432	54	444	Bone, Lymph node	Frameshift mutation	c.91C>T exon 2	^d^Pathogenic	Urine NE	^68^Ga-DOTATATE avid bone and lymph node lesions	Chronic autoimmune hepatitis	Breast cancer (grandmother)	No report	Recurrence and metastases after surgery with stability after receiving octreotide LAR 30 mg monthly
van der Tuin et al. (35)	3 out of 30, 10%	Dutch	Patient 24	50/F	Adrenal	Not report ed	60	120	Not reported	Nonsense point mutation	c.91C>T (p.Arg31*)	Pathogenic	NMN	Not reported	Negative	Negative	Not reported	Not reported
			Patient 27	23/M	Testis	Not reporter	26	36	Not reported	Nonsense point mutation	c.1534C>T (p.Arg512*)	Pathogenic	NMN, MTY	Not reported	Negative	PGL	Not reported	Not reported
			Patient 29	36/M	Retroperitoneum	Not reported	36	Not reported	Not reported	Nonsense point mutation	c.91C>T (p.Arg31*)	Pathogenic	NMN, MTY	Not reported	Negative	Negative	Not reported	Not reported

*CT, computed tomography; F, female; ^18^F-FDG, [^18^F]-fluorodeoxyglucose; ^68^Ga-DOTATATE, [^68^Ga]-(DOTA)-[Tyr3]-octreotate; M, male; MIBG, metaiodobenzylguanidine; MN, metanephrine; MTY, methoxytyramine; NA, not applicable; NE, norepinephrine; NMN, normetanephrine; PET, positron emission tomography; PHEO, pheochromocytoma; PGL, paraganglioma*.

a *Details from other patients with metastatic disease were not reported*.

b *Case report involving a patient with metastatic disease*.

c *There was not enough evidence to conclude that this variant is pathogenic since according to sorting intolerant from tolerant and polymorphism phenotyping techniques, it is probably benign but was not found in healthy controls*.

d *Authors presented data suggesting that mutations are pathogenic*.

All of the patients in our cohort were Caucasian. However, the ethnicity/race predilection of this condition remains inconclusive (12, 29, 31–35). Although the typical median age of PHEO/PGL detection in our cohort was 45 (range 11–57 years), it could be detected as young as 11. In other studies, age at detection was later, ranging from 18 to 50 years old (12, 29, 31–35). Male predominance in our cohort is similar to other studies (12, 31, 32, 34, 35) except in one study the only metastatic patient was female (33) and in another it was equal (29). Most patients presented with symptoms of catecholamine excess and a majority had extra-adrenal primary tumor, with the abdomen as the most common site, consistent to findings in other *SDHA* series (29, 32, 33, 35). The primary tumor of *SDHA*-related metastatic PHEO/PGL was found to be highly recurrent within a short period of time (median 12 months) after complete resection. It is reported that the large size of the primary tumor (>6.0 cm), its occurrence at sites other than the adrenal gland, recurrence, and multifocality of the lesions might indicate high metastatic potential of the tumor (36). In contrast, one series reported a varied recurrence after 12 and 432 months (29). The most common site of metastases was the bone which has also been reported in several other studies (29, 32).

*SDHx* mutations present with other tumors that are now considered to be a part of their characteristic syndromic presentation including *SDHx*-related PGL-RCC, Carney-Stratakis syndrome, Carney triad and PGL-pituitary adenoma (9, 37–39). As mentioned, an *SDHA* mutation has been associated with other disease entities. In one case, IHC for pituitary adenoma and RCC failed to show their direct association with *SDHA* (35). Likewise, in one of our patients, GIST and RCC were not related to *SDHA* mutations based on IHC. By contrast, in one study, 1 patient with germline *SDHA* mutation was found to be associated with GIST complicated by RCC (24), whereas in another study, germline *SDHA* mutation was found to be associated with GIST in 2 patients and neuroblastoma in 1 patient (20). Whether other tumors and autoimmune disorders described in this cohort and their family members are a part of syndromic presentation awaits confirmation. Due to a limitation of samples of other tumors, we do not know whether they could be associated with an *SDHA* mutation, and this warrants further investigation in the future.

Pathogenic germline non-sense mutations were found in more than half, with c.91C>T (p.Arg31^*^) as the most frequent gene locus. A similar finding has been reported in a nation-wide Dutch study (35), as well as in a report evaluating the pathogenicity and penetrance of *SDHA* variants reported in literature-based PHEO/PGL cases (40). Furthermore, in a particular case report, *SDHA*-related metastatic PGL was found to have truncating mutation at the said gene locus (33). These tumors tend to appear sporadic since all patients in our cohort lacked family history of PHEO/PGL, which also suggests its low penetrance. Previous studies have also reported that most of the patients lack a family history of PHEO/PGL (31–33, 35, 40). In the nation-wide Dutch study, most *SDHA*-related PHEO/PGLs, among the PGL-predisposing genes, had the lowest penetrance of 10% at the age of 70 (35). In another study, any-tumor penetrance at 40 years old among *SDHA* mutation carriers was 13% (31). The apparent low *SDHA* penetrance could be explained by the low frequency of loss of 5p15, which is the chromosomal region containing the *SDHA* locus unlike that of *SDHB* and *SDHD* loci (8, 10, 12, 22).

Biochemical phenotypes in this series are consistent with previous reports that described unique noradrenergic and/or dopaminergic biochemical phenotypes in *SDHx*-associated PHEO/PGL including *SDHA*-related PHEO/PGL (29, 33, 41–43). Our data suggest that plasma DA and MTY, and urine NE and NMN could be useful in the detection and surveillance of these tumors. The use of other biochemical tests including CgA in the initial detection and monitoring disease progression is limited in these patients in contrast to other *SDH* gene mutations (44).

In terms of functional imaging, ^123^I-MIBG scintigraphy and (^68^Ga-DOTATATE, ^18^F-FDOPA, ^18^F-FDG) PET/CT scans in many patients (*n* = 6/9, 66.67%) were performed at different time points, therefore direct comparison between ^123^I-MIBG scintigraphy and ^68^Ga-DOTATATE PET/CT scan was not possible. Nevertheless, ^18^F-FDG PET/CT scanning was performed during the time of ^123^I-MIBG scintigraphy so that these two modalities could be compared with each other. Based on these findings, it could be inferred that ^123^I-MIBG scintigraphy was not the optimal study for localizing tumor sites; further, since most of the *SDHA*-related metastatic PHEO/PGLs lacked avidity or detected few lesions on ^123^I-MIBG scintigraphy, it could not be included as a therapeutic option in this group of patients (33), which is similar to *SDHB*-related metastatic PHEOs/PGLs and points toward an unfavorable prognosis (45). The decreased tumor avidity for ^123^I-MIBG demonstrated by our patients could have been due to the poorly differentiated nature of these lesions. Poor performance of this type of scanning is more frequent for extra-adrenal tumors (45). Indeed, most of our patients had their tumors located in extra-adrenal sites. In contrast, ^68^Ga-DOTATATE PET/CT scan performed extremely well in these patients. Based on our results, we found that the functional imaging phenotype of *SDHA*-related PHEO/PGL is similar to *SDHx*-related PHEO/PGLs with the relation: ^68^Ga-DOTATATE>^18^F-FDG>^18^F-FDOPA>^123^I-MIBG. This finding is consistent with recent studies where ^68^Ga-DOTATATE PET/CT scanning was found superior to other anatomic and functional imaging modalities (^18^F-FDG, ^18^F-FDOPA, ^18^F-Fluorodopamine PET/CT, and CT and/or MRI scans) in the evaluation of *SDHB*-related metastatic PHEO/PGL (46). It is known that these tumors primarily overexpress somatostatin receptor type 2, and hence can be targeted by ^68^Ga-DOTATATE, a radiolabeled somatostatin analog for PET imaging. Recently, it was described that succinate is mainly responsible for the imaging phenotype of SDH-deficient PHEO/PGLs (47). Thus, low glucose uptake in some tumors reflects succinate levels in particular tumor tissue. Furthermore, the degree of dysfunctional oxidative phosphorylation and the expression of glucose transporters as well as the function of hexokinase could be other variables that may affect the final ^18^F-FDG uptake and retention of these tumors and superiority of ^68^Ga-DOTATATE over ^18^F-FDG PET/CT scan. In light of these findings, ^68^Ga-DOTATATE PET/CT scan is the imaging modality that is recommended in these patients. However, head-to-head comparison of per lesion detection rates is necessary in a larger cohort to validate these findings.

So far, the published literature available in PHEO/PGL evaluating the efficacy of cold somatostatin analogs is limited (48, 49). However, based on our experience, for inoperable primary and/or metastatic tumors, in the setting of a positive ^68^Ga-DOTATATE PET/CT scan, and those whom the use of radiation is contraindicated (children, a tumor close to very specific and important structures/nerves, previous external radiation or ^131^I-MIBG radiotherapy or PRRT, or personal beliefs refuting radiation), cold somatostatin analog therapy could be offered. In 2 large clinical trials, monthly administration of somatostatin analogs at anti-proliferative doses specifically, long-acting octreotide 30 milligrams (with significant reduction in CgA) monthly or lanreotide 120 milligrams (without significant improvement of symptoms and biochemical response) every 28 days prolonged the time to tumor progression of metastatic gastrointestinal neuroendocrine tumors (29, 50, 51). In our cohort, three patients (patients 2, 3, and 5) received cold somatostatin analog therapy. One patient (patient 2) received a short-acting octreotide at a dose much lower than used in the PROMID study (where long-acting octreotide was used) (51), and this did not result in a clinically meaningful response while the other patient (patient 5) had progression-free survival for 1.5 years with symptomatic and biochemical improvement after receiving lanreotide 120 mg every 14 days, protocol almost similar to that used in CLARINET FORTE study [ClinicalTrials.gov Identifier: NCT02651987] followed by radiation therapy which has showed response in an *SDHB*-related metastatic PHEO/PGL (52). On the other hand, patient 3 was started on lanreotide 120 mg every 28 days, protocol almost similar to that used in CLARINET study along with experimental ONC201 therapy and the patient has shown stable disease following 3 months of initiation of this combination therapy. ONC201 is a dopamine receptor inhibitor, DRD2, and is being evaluated as phase 2 clinical trial in neuroendocrine tumors including PHEO/PGLs [ClinicalTrials.gov Identifier: NCT03034200]. Propranolol is a non-selective beta-blocker which has shown anti-angiogenic and antitumor effects and is currently being repurposed in oncology (53, 54). However, whether somatostatin analog therapy had additive/synergistic effect when combined with other treatment(s) remains to be determined. These treatments show promise in *SDHA*-related metastatic PHEO/PGL and are worth studying to establish their efficacy and safety for this type of patients, especially those who present with inoperable tumors including metastatic disease. Moreover, it would be very important to compare long-acting octreotide vs. lanreotide in terms of treatment schedule, efficacy, long-term safety and outcome, and progression-free survival for these types of tumors. It would be interesting to determine how cold somatostatin analogs along with ^177^Lu-DOTATATE could work together, including appropriate treatment schedules. Lastly, the effect of metronomic schemes of chemotherapy for these types of tumors which was instituted in 2 patients (patients 5 and 10) warrants investigation as this mode of treatment has shown benefit to those with *SDHB*-related metastatic PGLs (55).

The *SDHA*-mutated tumors' response to radiotherapy and PRRT in this cohort was not consistent. Whether these treatment modalities have additive/synergistic effects when combined with other treatment(s) remains unclear, warranting additional studies to determine their effectiveness and safety among this group of patients.

Finally, after characterizing these patients, we realized the similarities of their disease characteristics with other *SDHx*-related PHEOs/PGLs. As with other *SDHx*-related PHEOs/PGLs, primary tumor of *SDHA*-related disease tends to occur at extra-adrenal locations and has a noradrenergic/dopaminergic biochemical phenotype (17, 42, 43, 56). Of note, *SDHA*-related metastatic PHEO/PGLs were found to have similarities with *SDHB*-related metastatic PHEO/PGL in terms of their tendency to have the primary tumor occur in extra-adrenal locations, site of metastases, exhibition of a noradrenergic/dopaminergic biochemical phenotype, avidity for ^68^Ga-DOTATATE on PET/CT, low ^123^I-MIBG avidity, and highly aggressive disease and poor treatment response (17, 41–43, 45, 46, 57–61). In our cohort, bone was the most common metastatic site followed by the lymph nodes, which is similar to that of *SDHB*-related metastatic PHEO/PGL (60–62). Compared with *SDHA*-related metastatic disease, the age at diagnosis among patients with *SDHB*-related metastatic disease was younger at 31 years old (61). These similarities between *SDHA*-related metastatic PHEOs/PGLs and *SDHB*-related metastatic PHEOs/PGLs may be due to the fact that *SDHA* gene mutations may affect SDHB function. In fact, it has been reported that when there is a defect in the *SDHA* gene, a defect in SDHB protein follows (34); patients with *SDHA* gene mutations lose both SDH subunits, namely SDHA and SDHB (34). One could hypothesize/speculate that in such a severe combination of having two dysfunctional subunits, natural selection would not allow these cells to survive or transform into cancer cells. Nevertheless, by some additional defense and other mechanisms (additional mutations, epigenetic changes), some cells indeed can transform into cancer cells and give rise to a neoplastic condition, in this case, PHEO/PGL. If they arise, then those tumors can be very problematic since they are missing two important proteins from the SDH complex and therefore, the Krebs cycle as well as oxidative phosphorylation might be deeply affected. Aggressive behavior and low penetrance of these tumors can be associated with the changes described above.

Few studies have analyzed genotype-phenotype relationships which are necessary for the clinician to make evidence-based decisions in terms of disease diagnosis and surveillance in regard to *SDHx* gene mutations (56). Therefore, evaluation of *SDHA* in patients with clinical presentations and a biochemical and functional imaging phenotype as described in this report may be of high importance in the early detection and management which may lead to better prognosis and outcomes (63). Although the disease has low penetrance and none of these patients had any family history of PHEOs/PGLs, we would recommend meticulous surveillance of the patients who already developed the disease. *SDHA*-related PHEO/PGL may not be well-detected by ^123^I-MIBG scintigraphy, is probably associated with other tumors, may be difficult to treat, and could have a rapidly progressive disease course. As evidenced in our cohort, it lacked features of hereditary disease that may serve as a clue to the clinician including young age at disease onset, bilaterality, multifocality, positive family history, and having syndromic characteristics and metastases (64–68). On the other hand, the *SDHA* carriers who are asymptomatic and have not developed PHEO/PGL are not likely to benefit from periodic surveillance screening due to the low penetrance of these tumors (40).

There are several limitations of this study. First, the study cohort is relatively small. However, considering the extreme rarity of metastatic *SDHA*-related PHEO/PGL, it becomes important to report findings from even a small study cohort. Second, it is partly a retrospective study and with our institution being an advanced tertiary research center, this study may have inherent biases including referral bias as the patients present to us or are referred at an advanced stage of the disease. Third, IHC was not available for all the coexisting tumors to determine their association with *SDHA*, and this warrants further investigation in the future as the information that may be obtained may potentially impact patient and family member screening and surveillance in the future. Fourth, ^123^I-MIBG scintigraphy and (^68^Ga-DOTATATE, ^18^F-FDOPA, ^18^F-FDG) PET/CT scans in three patients were performed at different time points, therefore direct comparison between ^123^I-MIBG scintigraphy and ^68^Ga-DOTATATE PET/CT scan was not possible. Finally, ^18^F-FDOPA PET/CT scan was not performed in all the patients and head-to-head per lesion comparison was not performed.

In conclusion, *SDHA*-related metastatic PHEO/PGLs behave like *SDHB*-related metastatic disease, showing aggressive behavior, similar imaging and biochemical phenotypes, and suboptimal responses to conventional treatments. Therefore, early detection, treatment, and surveillance are imperative for the affected patients. Further, larger studies are needed to uncover additional clinical aspects, other disease associations, and the most appropriate imaging, treatment, and surveillance techniques.

## Ethics Statement

All procedures performed in studies involving human participants were in accordance with the ethical standards of the institutional and/or national research committee and with the 1964 Helsinki declaration and its later amendments or comparable ethical standards.

## Informed Consent

Informed consent was obtained from the individuals who participated in this study or their legal guardians.

## Author Contributions

KP conceptualized the study. All authors were involved in the data gathering, narration of cases or appropriate discussion related to these cases, their presentations and explanation of results. AJ, KdL, CB, CM, AL, DT, and ACC reviewed the anatomic and functional imaging studies. All authors tabulated data. All authors made critical revision and approved the final version of the study.

### Conflict of Interest Statement

The authors declare that the research was conducted in the absence of any commercial or financial relationships that could be construed as a potential conflict of interest.
