# Supplementary material for: Clinical, Diagnostic, and Treatment Characteristics of SDHA-Related Metastatic Pheochromocytoma and Paraganglioma
Source: Front Oncol. 2019 Feb 22;9:53. doi: 10.3389/fonc.2019.00053 (PMC6395427; doi:10.3389/fonc.2019.00053)
Supplement: Supplementary file 1 [file Table_1.DOCX]

**Supplementary Table 1. Patients’ lesions throughout their disease course by anatomic and functional imaging.**

| **Patient number** | **Age (in years)** | **Anatomic Scan (CT/MRI)** | **^68^Ga-DOTATATE PET/CT** | **^18^F-FDG PET/CT or *PET/MRI** | **^18^F-FDOPA PET/CT** | **^123^I-MIBG or ^131^I-MIBG Scintigraphy** |
| --- | --- | --- | --- | --- | --- | --- |
| **1** | 11-12 | + | Not done | Not done | Not done | Negative |
| **12 PGL resection** | | | | | | |
|  | 12-14 | - | Not done | Not done | Not done | Not done |
|  | 15 | + | Not done | Suboptimal study | Not done | Negative |
|  | 16-18 | + | Not done | +^a^ | Not done | Not done |
|  | 21 | + | + | +^a^ | Not done | Not done |
| **21 PGL resection** | | | | | | |
|  | 22 | + | + | Not done | Not done | Not done |
| **2** | 53 | + | + | + | + | + |
| **53 PGL resection and short-acting octreotide** | | | | | | |
|  | 53-54 | + | Not done | Not done | Not done | Not done |
| **54 Received PRRT** | | | | | | |
|  | 54 | + | Not done | + | Not done | Not done |
| **55 CVD chemotherapy** | | | | | | |
|  | 55 | + | Not done | + | Not done | Not done |
| **55-56 Stopped CVD chemotherapy and tried bortezomib and clofarabine experimental therapy** | | | | | | |
|  | 56 | + | + | + | Not done | Not done |
| **3** | 14 | + | Not done | Not done | Not done | Not done |
| **14 PGL resection** | | | | | | |
|  | 14 | + | Not done | + | Not done | Negative |
|  | 15 | + | Not done | + | Not done | Not done |
|  | 15-16 | + | + | + | Not done | Not done |
| **16 PRRT** | | | | | | |
|  | 17 | + | + | + | Not done | Not done |
|  | 17 | + | Not done | + | Not done | Not done |
| **17-18 Lanreotide and ONC201 experimental therapy** | | | | | | |
|  | 18 | + | Not done | + | Not done | Not done |
| **4** | 53-57 | + | Not done | + | Not done | + |
| **57 PGL resection** | | | | | | |
|  | 57 | - | + | +^b^ | + | Not done |
|  | 58-59 | - | + | + | + | Not done |
|  | 61 | - | + | + | Not done | Not done |
| **5** | 53 | + | Not done | + | Not done | Not done |
| **53 PGL resection** | | | | | | |
|  | 57 | + | Not done | Not done | Not done | + |
| **57 Lanreotide and radiation therapy** | | | | | | |
|  | 59 | + | + | + | Not done | Not done |
| **59 Lanreotide and TMZ** | | | | | | |
|  | 59-61 | + | + | + | Not done | Not done |
| **6** | 20 | + | Not done | + | Not done | Not done |
| **20 PGL resection** | | | | | | |
|  | 21 | Negative | Not done | + | + | Not done |
|  | 22 | Negative | Not done | + | Not done | Not done |
|  | 23 | Not done | Negative | + | Not done | Not done |
|  | 24 | + | + | + | + |  |
|  | 25 | + | + | + | + | Not done |
| **7 56 Left adrenalectomy** | | | | | | |
|  | 66 | + | Not done | + | Not done | Not done |
| **66 Spinal PGL resection and radiation therapy** | | | | | | |
|  | 66 | + | Not done | Not done | Not done | Bone metastases |
| **66 ^131^I-MIBG therapy** | | | | | | |
|  | 66 | Not done | Not done | Not done | Not done | +^c^ |
|  | 67 | + | + | + | Not done | + |
| **CVD chemotherapy** | | | | | | |
|  | 68 | Not done | Not done | + | Not done | Not done |
| **8** | 29 | + | Not done | Not done | Not done | Not done |
| **29** **Angioembolization of T7 vertebral lesion** | | | | | | |
|  | 30 | + | Not done | - | Not done | Not done |
| **30** **Embolization, then partial excision of T7 lesion and stabilization of vertebra** | | | | | | |
|  | 31 | Not done | + | Not done | Not done | Not done |
|  | 32 | + | Not done | Not done | Not done | Not done |
| **32** **Embolization, partial excision of T7 lesion, and spinal cord decompression** | | | | | | |
|  | 32 | + | Not done | Not done | Not done | Not done |
| **32**  **Radiation therapy to T5-T9** | | | | | | |
|  | 32 | Not done | Not done | + | Not done | Not done |
|  | 32 | + | + | Not done | Not done | Not done |
|  | 33 | Not done | + | Not done | Not done | Not done |
|  | 33 | + | + | + | + | + |
| **33-34 PRRT** | | | | | | |
|  | 34 | + | + | + | + | Not done |
|  | 34 | + | + | + | + | Not done |
| **9** | 46 | + | Not done | Not done | Not done | Not done |
| **46 PGL resection** | | | | | | |
|  | 51 | + | Not done | Not done | Not done | Not done |
| **53 Radiation therapy and embolization of liver lesion** | | | | | | |
|  | 53 | + | + | + | + | + |
|  | 53 | + | Not done | + | Not done | Not done |
| **53-54 PRRT** | | | | | | |
|  | 54 | + | + | + | Not done | Not done |
| **54 Planned CVD chemotherapy** | | | | | | |
| **10** | 44 | + | Not done | Not done | Not done | Not done |
|  | 44 | Not done | Not done | Not done | Not done | + |
| **44 Cervical spine decompression, fusion, and radiation therapy to C-spine, ^131^I-MIBG therapy** | | | | | | |
|  | 45 | + | + | + | + | + |
| **45 TMZ** | | | | | | |

+, presence of pheochromocytoma/paraganglioma and/or its metastases; -, absence of pheochromocytoma/paraganglioma and/or its metastases

^a18^F-FDG PET/MRI was performed; ^b^Stomach (unclear significance and exact location); ^c131^I-MIBG scintigraphy was performed.

CT, Computed Tomography; CVD, combination chemotherapy with cyclophosphamide, vincristine, and dacarbazine; ^18^F-FDG, [^18^F]-fluorodeoxyglucose; ^18^F-FDOPA, [^18^F]-fluorodihydroxyphenylalanine; ^68^Ga-DOTATATE, [^68^Ga]-(DOTA)-[Tyr3]-octreotate; GIST, gastrointestinal stromal tumor; IVC, inferior vena cava; MIBG, metaiodobenzylguanidine; MRI, magnetic resonance imaging; PET, positron emission tomography; PGL, paraganglioma; PRRT, peptide receptor radionuclide therapy; TMZ, temozolomide.
